# *ABCB1* Genetic Variants as Predictors of Irinotecan-Induced Severe Gastrointestinal Toxicity in Metastatic Colorectal Cancer Patients

**DOI:** 10.3389/fphar.2020.00973

**Published:** 2020-06-30

**Authors:** Pau Riera, Alícia Artigas-Baleri, Juliana Salazar, Ana Sebio, Anna C. Virgili, María Jesús Arranz, David Páez

**Affiliations:** ^1^ Genetics Department, Hospital de la Santa Creu i Sant Pau, Barcelona, Spain; ^2^ Faculty of Pharmacy and Food Sciences, Universitat de Barcelona (UB), Barcelona, Spain; ^3^ Pharmacy Department, Hospital de la Santa Creu i Sant Pau, Barcelona, Spain; ^4^ Translational Medical Oncology Laboratory, Institut de Recerca Biomèdica Sant Pau, (IIB-Sant Pau), Barcelona, Spain; ^5^ Medical Oncology Department, Hospital de la Santa Creu i Sant Pau, Barcelona, Spain; ^6^ Research Laboratory, Fundació Docència i Investigació Mútua Terrassa, Terrassa, Spain; ^7^ U705, ISCIII Center for Biomedical Research on Rare Diseases (CIBERER), Barcelona, Spain

**Keywords:** *ABCB1*, P-glycoprotein, irinotecan (CPT-11), gastrointestinal toxicity, diarrhea, mucositis, biomarker, colorectal cancer

## Abstract

Irinotecan is widely used in the treatment of metastatic colorectal cancer (mCRC) despite its severe toxicities. Toxicity is often associated with the UGT1A1*28/*28 genotype. An explanation for idiopathic toxicity beyond the *UGT1A1* biomarker, however, remains a major concern for clinicians. One of the main irinotecan transporters is P-glycoprotein (P-gp), which is a hepatic efflux pump encoded by *ABCB1*. P-gp is involved in the biliary excretion of irinotecan and its active metabolite SN-38. We aimed to assess whether functional variants in *ABCB1* also contribute to identifying patients at risk of toxicity. A cohort of 308 mCRC patients treated with irinotecan-based regimens were genotyped for polymorphisms in *ABCB1* (rs1128503, rs2032582, and rs1045642). The effect of these variants and their haplotypes on irinotecan-induced severe toxicity (diarrhea, neutropenia, asthenia, nausea, and mucositis) was assessed. After adjusting for the relevant clinical and pathological parameters in the multivariate analysis, we found rs1128503 was significantly associated with severe diarrhea and mucositis (*P*=0.014 and *P*=0.002, respectively). Additionally, rs2032582 was associated with severe mucositis (*P*<0.001). Our results show that rs1128503 genotyping could help to predict severe gastrointestinal toxicity induced by irinotecan.

## Introduction

Irinotecan (CPT-11) is an antitumor agent that is broadly used in metastatic colorectal cancer (mCRC) patients and normally co-administered with infusional 5-fluorouracil/leucovorin (FOLFIRI regimen). Its use is hampered by toxicities such as severe diarrhea and neutropenia in more than one-third of the patients (Fuchs et al., 2003).

In the liver, CPT-11 is converted to 7-ethyl-10-hydroxy-camptothecin (SN-38), which is responsible for both the efficacy and the toxicity of the drug. SN-38 is mainly eliminated through glucuronidation by the uridine 5′-diphospho-(UDP)-glucuronosyltransferase (UGT) isoform 1A1, which is encoded by the *UGT1A1* gene. The presence of seven TA repeats (UGT1A1*28 allele) in the promoter region of this gene reduces enzymatic activity (Bosma et al., 1995) and patients homozygous for this variant have a higher risk of irinotecan-induced toxicity (Innocenti et al., 2004; Marcuello et al., 2004; Rouits et al., 2004; Riera et al., 2018). However, *UGT1A1* genetic status does not always explain severe toxicity, suggesting that other mechanisms contribute to the appearance of side effects.

An ATP-powered pump encoded by the *ABCB1* gene, P-glycoprotein (P-gp), participates in the biliary excretion of CPT-11 and SN-38. The enhancement of P-gp expression increases biliary secretion of SN-38 and reduces its plasma concentrations, conferring a higher risk of intestinal toxicity but a lower risk of neutropenia (Iyer et al., 2002; Mathijssen et al., 2003; Han et al., 2009; Filipski et al., 2014; Li et al., 2018). The most well-known *ABCB1* variants, rs1128503 (1236 C>T), rs2032582 (2677 G>T/A), and rs1045642 (3435 C>T), modulate P-gp expression (Hoffmeyer et al., 2000; Haenisch et al., 2007), CPT-11 and SN-38 plasma concentrations, and renal clearance (Mathijssen et al., 2003; Sai et al., 2003). However, the influence of these variants on irinotecan-induced severe toxicity remains inconclusive (Han et al., 2007; Innocenti et al., 2009; Glimelius et al., 2011; Teft et al., 2015; Li et al., 2018; Salvador-Martín et al., 2018).

Based on the relevance of P-gp on irinotecan pharmacokinetics, we conducted a study in a cohort of mCRC patients treated with an irinotecan-containing therapy in whom the *UGT1A1* gene had previously been analyzed. The aim was to elucidate whether the aforementioned *ABCB1* variants could also help to identify patients who develop severe irinotecan-induced toxicity.

## Material and Methods

### Study Population

We retrospectively included patients from Hospital de la Santa Creu i Sant Pau (HSCSP, Barcelona, Spain) who were diagnosed between 2001 and 2016 with mCRC and treated with an irinotecan-containing regimen. All patients had an Eastern Cooperative Oncology Group (ECOG) performance status ≤2 and age ≥ 18. Irinotecan-containing regimens were biweekly FOLFIRI (irinotecan 180 mg/m^2^), irinotecan in monotherapy every 3 weeks (350 mg/m^2^, except for one patient with the UGT1A1*28/*37 genotype who received a reduced dose of 150 mg/m^2^), and other regimens containing irinotecan (irinotecan 125–350 mg/m^2^). We excluded those patients without available germline DNA or without all the toxicities properly collected. The toxicities assessed were diarrhea, neutropenia, asthenia, nausea, and mucositis. For each side-effect, we recorded the highest grade of toxicity experienced by each patient. Toxicities were graded (1 to 4) on the basis of the Common Terminology Criteria for Adverse Events v5.0 (CTCAE) of the National Cancer Institute. Grade 3 to 4 toxicities were considered severe toxicities. The study was approved by the Institutional Ethics Committee at HSCSP and written informed consent was obtained from all participants.

### Genotyping

Germline DNA was extracted from blood cells by the salting-out procedure (Miller et al., 1988). Three functional *ABCB1* gene polymorphisms, rs1128503 (1236 C>T), rs2032582 (2677 G>T/A), and rs1045642 (3435 C>T), were genotyped using TaqMan PCR assays (Applied Biosystems, Foster City, CA, USA). Due to the rs2032582 triallelic condition, two probes (C_11711720C_30 and C_11711720D_40) were needed to genotype this variant. The other probes used were C_7586662_10 for rs11128503, and C_7586657_20 for rs1045642. Genotyping was performed on a 7500 Real Time PCR System (Applied Biosystems, Foster City, CA, USA).

### Statistics

Allele frequencies for each *single nucleotide polymorphism* (SNP) were checked by direct counting and compared to published data (1000 Genomes Project Consortium et al., 2015). Genotype data were tested for Hardy-Weinberg equilibrium using chi-squared tests. The triallelic condition of the rs2032582 variant results in six possible genotypes (GG, GT, GA, TT, TA, and AA). To simplify the statistical analyses, we combined patients with GT or GA genotypes, and also patients with TT, TA, or AA genotypes. For haplotype analysis, GA, TA, and AA patients were excluded, as PLINK removes triallelic SNP genotype calls. We performed phased haplotype analyses (using the E-M algorithm) including the three *ABCB1* variants or using the window option for diplotype analyses. Associations between toxicities and genetic variants/haplotypes were measured using chi-squared tests. Multivariate analyses were carried out, including sex, age, chemotherapy treatment, line of chemotherapy, and *UGT1A1* and *ABCB1* genotypes. *P*-values <0.05 were considered statistically significant. Bonferroni correction for multiple comparisons was applied (*P*-values <2.4·10^−4^). All statistical analyses were conducted using SPSS (v25.0, IBM) and PLINK (v1.07, Shaun Purcell).

## Results

### Patient Population

We studied a total of 308 patients. Most patients (n=283) were treated with irinotecan in combination with 5-fluorouracil and leucovorin (FOLFIRI). A total of 176 patients had been enrolled in clinical trials (Marcuello et al., 2011; Páez et al., 2019). The presence of the UGT1A1*28, UGT1A1*37, and UGT1A1*36 alleles was determined in a previous study (Riera et al., 2018). **Table 1** shows patients’ clinical characteristics along with *UGT1A1* genotype status. **Supplementary Table 1** shows toxicity grades according to CTCAE v5.0.

**Table 1:** Baseline patient characteristics (*n*=308).

		n		%
Sex Male Female Age Mean Range	63 24–83	194 114	63.0% 37.0%	
Primary tumor site Right colon Left colon		76 232	24.7% 75.3%	
Chemotherapy treatment FOLFIRI Irinotecan monotherapy Other irinotecan combinations		283 13 12	91.9% 4.2% 3.9%	
Line of chemotherapy First line ≥ Second line *UGT1A1* genotype UGT1A1 *1/*1 UGT1A1 *1/*28 UGT1A1 *28/*28 UGT1A1 *28/*37		244 64 136 143 28 1	79.2% 20.8% 44.2% 46.4% 9.1% 0.3%	

FOLFIRI, combination of irinotecan, 5-fluorouracil, and leucovorin, UGT1A1, uridine 5′-diphospho-(UDP)-glucuronosyltransferase (UGT) isoform 1A1

### Genetic Studies

Allelic frequencies for all polymorphisms were within the probability limits of Hardy–Weinberg equilibrium (*P*>0.05), and their minor allele frequencies were in accordance with European population frequencies reported in the 1000 Genomes Project (1000 Genomes Project Consortium et al., 2015). Genotyping was successful in 307 patients (99.7%).

#### Associations Between *ABCB1* Variants and Severe Side Effects

**Figure 1:**
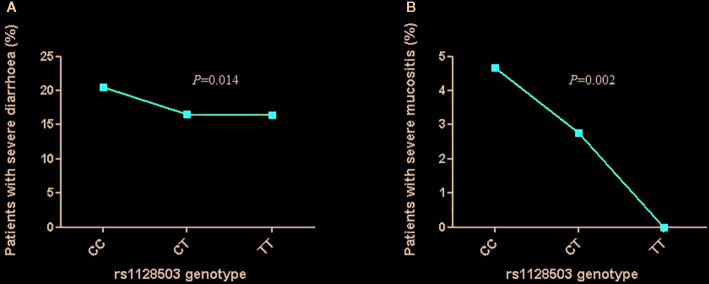
Association between rs1128503 genotype and irinotecan-induced gastrointestinal severe toxicities [**(A)** diarrhea; **(B)** mucositis)].

In the univariate analysis, we found no significant associations between the tested *ABCB1* variants and the presence of irinotecan-induced side effects. In the multivariate analysis, rs1128503 was significantly associated with severe diarrhea and mucositis (*P*=0.014 and *P*=0.002, respectively). Patients harboring the CC genotype for rs1128503 presented a higher prevalence of severe diarrhea and mucositis than those with CT and TT genotypes (**Figure 1**). In addition, rs2032582 was associated with severe mucositis (*P*=0.0001). This association retained its significance after Bonferroni correction. **Table 2** shows the genotyping distributions and associations between the three polymorphisms and the toxicities assessed. When analyzing gene interactions, we found no significant interactions between the *UGT1A1* and the *ABCB1* variants tested.

**Table 2:** Univariate and multivariate associations between *ABCB1* genotypes and grade 3–4 toxicities.

Genotype	n (%)	Diarrhea, n (%)	Neutropenia, n (%)	Asthenia, n (%)	Nausea, n (%)	Mucositis, n (%)
**rs1128503 (1236 C>T)**						
** CC**	107 (34.9%)	22 (20.6%)	29 (27.1%)	24 (22.4%)	10 (9.4%)	5 (4.7%)
** CT**	145 (47.2%)	24 (16.6%)	27 (18.6%)	29 (20.0%)	11 (7.6%)	4 (2.8%)
** TT**	55 (17.9%)	9 (16.4 %)	11 (20.0%)	11 (20.0%)	6 (10.9%)	0 (0%)
*** P-value (univariate analysis)***		*P* = 0.676	*P* = 0.256	*P* = 0.883	*P* = 0.737	*P* = 0.245
*** P-value (multivariate analysis)***		***P* = 0.014**	*P* = 0.990	*P* = 0.236	*P* = 0.615	***P* = 0.002**
**rs2032582 (2677 G>T/A)**						
** GG**	102 (33.2%)	18 (17.7%)	28 (27.5%)	20 (19.6%)	8 (7.8%)	2 (2.0%)
** GT**	144 (46.9%)					
** GA**	7 (2.3%)					
** GT+GA**		26 (17.2%)	28 (18.5%)	33 (21.9%)	14 (9.3%)	5 (3.3%)
** TT**	51 (16.6%)					
** TA**	3 (1.0%)					
** TT+TA**		11 (20.4%)	11 (20.4%)	11 (20.4%)	5 (9.3%)	2 (3.7%)
*** P-value (univariate analysis)***		*P* = 0.871	*P* = 0.233	*P* = 0.907	*P* = 0.917	*P* = 0.768
*** P-value (multivariate analysis)***		*P* = 0.095	*P* = 0.213	*P* = 0.586	*P* = 0.713	***P* = 0.0001** ^a^
**rs1045642 (3435 C>T)**						
** CC**	90 (29.3%)	15 (16.7%)	24 (26.7%)	16 (17.8%)	6 (6.7%)	3 (3.3%)
** CT**	149 (48.5%)	25 (16.8%)	27 (18.1%)	34 (22.8%)	15 (10.1%)	6 (4.0%)
** TT**	68 (22.2%)	15 (22.1%)	16 (23.5%)	14 (20.6%)	6 (8.8%)	0 (0%)
*** P-value (univariate analysis)***		*P* = 0.600	*P* = 0.279	*P* = 0.648	*P* = 0.667	*P* = 0.255
*** P-value (multivariate analysis)***		*P* = 0.197	*P* = 0.207	*P* = 0.240	*P* = 0.659	*P* = 0.352

The bold values indicate the statistically significant P-values (P < 0.05).

^a^Significant after Bonferroni correction (P < 2.4·10^−4^).

ABCB1, ATP binding cassette subfamily B member 1.

#### Associations Between *ABCB1* Haplotypes and Severe Side Effects

In the univariate analysis, we found that the diplotypes rs1045642T-rs1128503C (frequency=10.5%) and rs1128503C-rs2032582T (frequency=1.9%) were associated with a higher risk of diarrhea (*P*=0.034 and *P*=0.002, respectively). The rs1128503C-rs2032582T diplotype was also associated with a higher risk of mucositis (*P*=3.8·10^−8^), asthenia (*P*=0.043), and nausea (*P*=0.034). The association with mucositis remained statistically significant after applying Bonferroni test.

In the multivariate analysis, the associations of both diplotypes with diarrhea remained significant (*P*=0.035 for rs1045642T-rs1128503C and *P*=0.005 for rs1128503C-rs2032582T). The associations of the diplotype rs1128503C-rs2032582T with mucositis and nausea also retained their significance after multivariate adjustments (*P*=0.00018 and *P*=0.049, respectively), but only the association with mucositis retained its significance after Bonferroni correction. No other associations between *ABCB1* diplotypes and the occurrence of severe side effects were found. These results are shown in the **Supplementary Table 2**.

The haplotype analyses considering the three *ABCB1* gene variants (rs1128503-rs2032582-rs1045642) identified seven of the eight possible haplotypes in our cohort of patients. The most frequent haplotypes were CGC and TTT, with a prevalence of 47.5% (n=282) and 37.4% (n=222), respectively (**Table 3**). In contrast, the haplotype CTC was rare (0.3%) and the TGT haplotype was not harbored by any patient.

**Table 3:** Univariate and multivariate associations between *ABCB1* haplotypes and grade 3–4 toxicities.

		Haplotype (rs1128503-rs2032582-rs1045642)*					
		TTT (n=222)	TTC (n=13)	CTT (n=9)	TGC (n=16)	CGT (n=50)	CGC (n=282)
**Diarrhoea**	Unaffected (n=485) Affected (n=107) *P-value* (univariate analysis) *P-value* (multivariate analysis)	185 (38.1%) 37 (34.6%) 0.511 0.459	10 (2.1%) 3 (2.8%) 0.703 0.888	4 (0.8%) 5 (4.7%) **0.005** **0.012**	15 (3.1%) 1 (0.9%) 0.213 0.193	38 (7.8%) 12 (11.2%) 0.256 0.306	233 (48.0%) 49 (45.8%) 0.680 0.791
**Neutropenia**	Unaffected (n=458) Affected (n=134) *P-value* (univariate analysis) *P-value* (multivariate analysis)	179 (39.1%) 43 (32.1%) 0.151 0.122	10 (2.2%) 3 (2.2%) 0.963 0.631	5 (1.1%) 4 (3.0%) 0.140 0.234	13 (2.8%) 3 (2.2%) 0.708 0.815	38 (8.3%) 12 (9.0%) 0.812 0.361	213 (46.5%) 69 (51.5%) 0.303 0.408
**Asthenia**	Unaffected (n=468) Affected (n=124) *P-value* (univariate analysis) *P-value* (multivariate analysis)	176 (37.6%) 46 (37.1%) 0.935 0.975	12 (2.6%) 1 (0.8%) 0.246 0.203	4 (0.9%) 5 (4.0%) **0.015** **0.047**	13 (2.8%) 3 (2.4%) 0.829 0.979	40 (8.5%) 10 (8.1%) 0.901 0.765	223 (47.6%) 59 (47.6%) 0.984 0.777
**Nausea**	Unaffected (n=538) Affected (n=54) *P-value* (univariate analysis) *P-value* (multivariate analysis)	201 (37.4%) 21 (38.9%) 0.812 0.799	13 (2.4%) 0 (0%) 0.263 0.708	6 (1.1%) 3 (5.6%) **0.014** **0.029**	14 (2.6%) 2 (3.7%) 0.633 0.753	47 (8.7%) 3 (5.6%) 0.447 0.427	257 (47.8%) 25 (46.3%) 0.832 0.866
**Mucositis**	Unaffected (n=579) Affected (n=13) *P-value* (univariate analysis) *P-value* (multivariate analysis)	219 (37.8%) 3 (23.1%) 0.214 0.251	13 (2.2%) 0 (0%) 0.906 0.921	7 (1.2%) 2 (15.4%) **8.8·10^−6a^** **0.002**	16 (2.8%) 0 (0%) 0.541 0.998	50 (8.6%) 0 (0%) 0.302 0.458	274 (47.3%) 8 (61.5%) 0.353 0.733

*The haplotype CTC was present in two patients. Due to its low frequency, it was excluded from the statistical analysis. The haplotype TGT was not harbored by any patient.

^a^Significant after Bonferroni correction (P < 2.4·10^-4^).

The bold values indicate the statistically significant P-values (P < 0.05).

ABCB1, ATP binding cassette subfamily B member 1.

In the univariate analysis, the CTT haplotype (frequency= 1.5%) was significantly associated with a higher risk of severe diarrhea, mucositis, asthenia, and nausea (*P*=0.005, *P*=8.8·10^−6^, *P*=0.015, and *P*=0.014, respectively). These associations retained their significance after multivariate adjustments (*P*=0.012, *P*=0.002, *P*=0.047, and *P*=0.029, respectively) (see **Table 3**). Only the association with mucositis in the univariate analysis remained statistically significant after applying the Bonferroni test.

## Discussion

This study shows the *ABCB1* rs1128503 variant is a promising predictor of irinotecan-induced severe gastrointestinal toxicity, in particular diarrhea and mucositis. This result is reinforced by the significant associations found between the diplotypes and haplotypes containing the rs1128503-C allele and the referred toxicities. The rs2032582 variant also appears to be associated with severe mucositis.

Irinotecan-induced toxicities, the most common being neutropenia and diarrhea, usually lead to dose reduction or treatment withdrawal (Douillard et al., 2000). The Food and Drug Administration (FDA) drug label for irinotecan includes therapeutic recommendations for UGT1A1*28 homozygous patients to reduce the risk of developing neutropenia [Irinotecan (CAMPTOSAR) drug label. Food and Drug Administration. (Accession date: 24/02/2020)]. However, no recommendations are given to reduce the likelihood of irinotecan-induced severe gastrointestinal toxicity. The variability observed in the development of toxicity in clinical practice suggests that other polymorphisms or haplotypes could be involved. A growing body of evidence shows that variants in CPT-11 transporter genes could be helpful to identify patients at risk of severe side effects (Innocenti et al., 2009; Chen et al., 2015; Teft et al., 2015). *ABCB1* gene is one of the most promising candidates, considering the relevance of the P-gp on the biliary secretion of irinotecan and its metabolites from the liver (Iyer et al., 2002). Great attention has been paid to rs1128503 (1236 C>T), rs2032582 (2677 G>T/A), and rs1045642 (3435 C>T), variants found to be in strong linkage disequilibrium and associated with drug exposure (Mathijssen et al., 2003; Han et al., 2007; Li et al., 2018).

Rhodes et al. analyzed 11 polymorphisms in 54 patients with metastatic colorectal cancer, and reported significant associations with grade 3–4 toxicity and grade 3–4 neutropenia when combining *ABCB1*, C1236T, and *SLCO1B1* T521C polymorphisms (Rhodes et al., 2007). More recently, Salvador-Martín et al. analyzed the association between variants in ABC efflux transporter genes and irinotecan adverse effects, and found that the *ABCB1* variants were significantly associated with overall toxicity (*P*<0.01) and with higher hematological toxicity including, but not limited to, neutropenia (*P*<0.01) (Salvador-Martín et al., 2018). However, other studies conducted in mCRC patients treated with irinotecan in whom *ABCB1* variants were analyzed did not show significant associations with severe neutropenia (De Mattia et al., 2013; Chen et al., 2015; Teft et al., 2015; Yan et al., 2016). Our findings support these negative results as we found no associations with severe neutropenia in the 307 patients analyzed. These data, together with the significant association obtained with the UGT1A1*28 marker (*P *= 0.037) in a previous study by our group (Riera et al., 2018), lead us to infer that *UGT1A1* is a more powerful predictor of severe neutropenia than *ABCB1*.

Few studies have analyzed *ABCB1* variants as determinants of gastrointestinal toxicity in mCRC patients treated with irinotecan, and most of them produced negative results (De Mattia et al., 2013; Chen et al., 2015; Teft et al., 2015; Yan et al., 2016; Salvador-Martín et al., 2018). Nevertheless, in a cohort of 56 mCRC patients receiving an irinotecan-based treatment, Cortejoso et al. found that patients with the CC genotype for the rs1045642 variant had a higher probability of diarrhea than patients with CT and TT genotypes (*P*=0.039) (Cortejoso, et al., 2013). In line with this study, and after adjusting for clinicopathological covariates and the UGT1A1*28 genotype in a larger sample, we found that patients with the CC genotype for the *ABCB1* rs1128503 variant were at highest risk of diarrhea (*P*=0.014) and mucositis (*P*=0.002). Furthermore, diplotypes and haplotypes including the rs1128503-C allele were significantly associated with severe gastrointestinal toxicity, suggesting this allele contributes to the risk of toxicity. Of note, Mathijssen et al. reported that patients homozygous for the rs1128503-T allele showed greater exposure to both irinotecan (*P*=0.038) and SN-38 (*P*=0.031) than the other patients in the trial (Mathijssen et al., 2003). They also postulated that this synonymous variant could alter RNA stability and affect the expression of ABCB1. Thus, in keeping with our findings, we speculate that patients carrying the TT genotype may present less biliary excretion and a lower risk of gastrointestinal toxicity.

In an attempt to improve the pharmacogenetic prediction of irinotecan-induced severe toxicity, we focused our study on the three most informative *ABCB1* variants. Although this approach allowed us to achieve a higher statistical power, it has the limitation of losing the genetic effect of other variants located in the *ABCB1* gene or in other hepatic efflux transporters, such as *ABCC2* or *ABCG2*. A second limitation of our study could be the similar side-effect profile of 5-fluorouracil and irinotecan. However, the ABCB1 transporter has not been found to be involved in the pharmacokinetics of 5-fluorouracil, suggesting that the associations observed are likely due to irinotecan effects. A third limitation is that we did not record whether toxicity appeared in the first or later cycles or whether diarrhea was early-onset or delayed. Neither did we record the prophylactic anti-diarrheal agents received by each patient. Taken together, these data would have been useful to interpret the results. Finally, the associations found in this study should be considered exploratory because only the results with a *P*<0.001 survived Bonferroni correction for multiple comparisons. We note, however, that the Bonferroni correction was too conservative as the *ABCB1* variants were in linkage disequilibrium and the procedure ignored the dependencies between these variants. This limitation could be overcome by validating our findings in a similar and independent cohort of patients, and also by performing functional studies.

In conclusion, our findings in a large and homogeneous cohort of mCRC patients show that rs1128503 and the diplotypes/haplotypes containing the C allele of this variant could be useful predictors of irinotecan-induced severe gastrointestinal toxicity. Further studies are needed to increase evidence regarding the utility of the rs1128503 variant in clinical practice.

## Data Availability Statement

The raw data supporting the conclusions of this article will be made available by the authors, without undue reservation.

## Ethics Statement

The studies involving human participants were reviewed and approved by Institutional Ethics Committee at Hospital de la Santa Creu i Sant Pau. The patients/participants provided their written informed consent to participate in this study.

## Author Contributions

PR, AA-B, JS, and DP contributed to the conception and design of the study. PR, AA-B, and JS performed genotyping and collected data. AS and AV collected data. PR, AA-B, JS, MA, and DP analyzed the data and performed statistical analyses. PR, AA-B, JS, and DP wrote the manuscript. All authors contributed to the article and approved the submitted version.

## Conflict of Interest

AS reports receiving travel grants from Merck, Amgen, Sanofi, and Roche. AV has honoraria from Speakers Bureau of Amgen, Sanofi, and Kyowa Kirin, declares a scientific advisory role for Roche and Amgen, and reports receiving travel grants from Merck, Roche, Amgen, Sanofi, MSD, and Servier. DP has honoraria from Speakers Bureau of Merck Serono and F. Hoffmann-La Roche Ltd, and declares a scientific advisory role for Amgen and Sanofi.

The remaining authors declare that the research was conducted in the absence of any commercial or financial relationships that could be construed as a potential conflict of interest.
