# Supplementary material for: ABCB1 Genetic Variants as Predictors of Irinotecan-Induced Severe Gastrointestinal Toxicity in Metastatic Colorectal Cancer Patients
Source: Front Pharmacol. 2020 Jun 30;11:973. doi: 10.3389/fphar.2020.00973 (PMC7338599; doi:10.3389/fphar.2020.00973)
Supplement: Supplementary file 1 [file Table_1.docx]

| **Supplementary table 1. Prevalence and grade of the toxicities assessed according to CTCAE (n=308)** | | | | | |
| --- | --- | --- | --- | --- | --- |
| **Toxicity** | **Grade 0, n (%)** | **Grade 1, n (%)** | **Grade 2, n (%)** | **Grade 3, n (%)** | **Grade 4, n (%)** |
| Diarrhoea | 131 (42.5%) | 58 (18.8%) | 64 (20.8%) | 52 (16.9%) | 3 (1.0%) |
| Neutropenia | 150 (48.7%) | 20 (6.5%) | 70 (22.7%) | 57 (18.5%) | 11 (3.6%) |
| Asthenia | 108 (35.1%) | 45 (14.6%) | 90 (29.2%) | 61 (19.8%) | 4 (1.3%) |
| Nausea | 170 (55.2%) | 41 (13.3%) | 70 (22.7%) | 27 (8.8%) | 0 (0%) |
| Mucositis | 214 (69.5%) | 44 (14.3%) | 41 (13.3%) | 9 (2.9%) | 0 (0%) |

Abbreviations: CTCAE, Common Terminology Criteria for Adverse Events (v5.0)
