# Supplementary material for: ABCB1 Genetic Variants as Predictors of Irinotecan-Induced Severe Gastrointestinal Toxicity in Metastatic Colorectal Cancer Patients
Source: Front Pharmacol. 2020 Jun 30;11:973. doi: 10.3389/fphar.2020.00973 (PMC7338599; doi:10.3389/fphar.2020.00973)
Supplement: Supplementary file 2 [file Table_2.docx]

| **Supplementary table 2.**  Univariate and multivariate associations between *ABCB1* diplotypes and grade 3-4 toxicities | | | | | | | | | |
| --- | --- | --- | --- | --- | --- | --- | --- | --- | --- |
|  | | **rs1045642-rs1128503** | | | | **rs1128503-rs2032582** | | | |
|  |  | **TT**  **(n=219)** | **CT**  **(n=32)** | **TC**  **(n=62)** | **CC**  **(n=281)** | **TT**  **(n=235)** | **CT**  **(n=11)** | **TG**  **(n=16)** | **CG**  **(n=332)** |
| **Diarrhoea** | Unaffected (n=486)  Affected (n=108)  *P-value* (univariate analysis)  *P-value* (multivariate analysis) | 182 (37.4%)  37 (34.3%)  0.482  0.464 | 28 (5.8%)  4 (3.7%)  0.497  0.317 | 45 (9.3%)  17 (15.7%)  **0.034**  **0.035** | 231 (47.5%)  50 (46.3%)  0.756  0.932 | 195 (40.1%)  40 (37.0%)  0.553  0.498 | 5 (1.0%)  6 (5.6%)  **0.002**  **0.005** | 15 (3.1%)  1 (0.9%)  0.210  0.193 | 271 (55.8%)  61 (56.5%)  0.892  0.781 |
| **Neutropenia** | Unaffected (n=460)  Affected (n=134)  *P-value* (univariate analysis)  *P-value* (multivariate analysis) | 176 (38.3%)  43 (32.1%)  0.163  0.125 | 26 (5.7%)  6 (4.5%)  0.741  0.812 | 46 (10.0%)  16 (11.9%)  0.429  0.174 | 212 (46.1%)  69 (51.5%)  0.311  0.428 | 189 (41.1%)  46 (34.3%)  0.159  0.097 | 7 (1.5%)  4 (3.0%)  0.269  0.358 | 13 (2.8%)  3 (2.2%)  0.712  0.815 | 251 (54.6%)  81 (60.4%)  0.228  0.192 |
| **Asthenia** | Unaffected (n=470)  Affected (n=124)  *P-value* (univariate analysis)  *P-value* (multivariate analysis) | 174 (37.0%)  45 (36.3%)  0.944  0.984 | 27 (5.7%)  5 (4.0%)  0.358  0.356 | 46 (9.8%)  16 (12.9%)  0.378  0.215 | 223 (47.4%)  58 (46.8%)  0.955  0.743 | 188 (40.0%)  47 (37.9%)  0.671  0.704 | 6 (1.3%)  5 (4.0%)  **0.043**  0.083 | 13 (2.8%)  3 (2.4%)  0.832  0.979 | 263 (56.0%)  69 (55.6%)  0.950  0.896 |
| **Nausea** | Unaffected (n=540)  Affected (n=54)  *P-value* (univariate analysis)  *P-value* (multivariate analysis) | 198 (36.7%)  21 (38.9%)  0.807  0.804 | 30 (5.6%)  2 (3.7%)  0.684  0.628 | 56 (10.4%)  6 (11.1%)  0.770  0.764 | 256 (47.4%)  25 (46.3%)  0.817  0.846 | 214 (39.6%)  21 (38.9%)  0.916  0.931 | 8 (1.5%)  3 (5.6%)  **0.034**  **0.049** | 14 (2.6%)  2 (3.7%)  0.631  0.753 | 304 (56.3%)  28 (51.9%)  0.531  0.558 |
| **Mucositis** | Unaffected (n=580)  Affected (n=14)  *P-value* (univariate analysis)  *P-value* (multivariate analysis) | 216 (37.2%)  3 (21.4%)  0.207  0.299 | 32 (5.5%)  0 (0%)  0.427  0.620 | 60 (10.3%)  2 (14.3%)  0.579  0.524 | 272 (46.9%)  9 (64.3%)  0.215  0.374 | 232 (40.0%)  3 (21.4%)  0.160  0.251 | 8 (1.4%)  3 (21.4%)  **3.8·10^-8 a^**  **0.00018^a^** | 16 (2.8%)  0 (0%)  0.529  0.998 | 324 (55.9%)  8 (57.1%)  0.924  0.815 |

|  | | | | | |
| --- | --- | --- | --- | --- | --- |
|  | | **rs2032582-rs1045642** | | | |
|  |  | **TT**  **(n=230)** | **GT**  **(n=51)** | **TC**  **(n=16)** | **GC**  **(n=297)** |
| **Diarrhoea** | Unaffected (n=486)  Affected (n=108)  *P-value* (univariate analysis)  *P-value* (multivariate analysis) | 188 (38.7%)  42 (38.9%)  0.992  0.991 | 39 (8.0%)  12 (11.1%)  0.279  0.318 | 12 (2.5%)  4 (3.7%)  0.426  0.528 | 247 (50.8%)  50 (46.3%)  0.379  0.472 |
| **Neutropenia** | Unaffected (n=460)  Affected (n=134)  *P-value* (univariate analysis)  *P-value* (multivariate analysis) | 183 (39.8%)  47 (35.1%)  0.310  0.229 | 39 (8.5%)  12 (9.0%)  0.820  0.372 | 13 (2.8%)  3 (2.2%)  0.786  0.517 | 225 (48.9%)  72 (53.7%)  0.342  0.367 |
| **Asthenia** | Unaffected (n=470)  Affected (n=124)  *P-value* (univariate analysis)  *P-value* (multivariate analysis) | 179 (38.1%)  51 (41.1%)  0.570  0.553 | 41 (8.7%)  10 (8.1%)  0.886  0.785 | 15 (3.2%)  1 (0.8%)  0.197  0.176 | 235 (50.0%)  62 (50.0%)  0.960  0.793 |
| **Nausea** | Unaffected (n=540)  Affected (n=54)  *P-value* (univariate analysis)  *P-value* (multivariate analysis) | 206 (38.1%)  24 (44.4%)  0.384  0.381 | 48 (8.9%)  3 (5.6%)  0.442  0.419 | 16 (3.0%)  0 (0%)  0.246  0.486 | 270 (50.0%)  27 (50.0%)  0.972  0.957 |
| **Mucositis** | Unaffected (n=580)  Affected (n=14)  *P-value* (univariate analysis)  *P-value* (multivariate analysis) | 225 (38.8%)  5 (35.7%)  0.798  0.993 | 51 (8.8%)  0 (0%)  0.263  0.619 | 15 (2.6%)  1 (7.1%)  0.274  0.224 | 289 (49.8%)  8 (57.1%)  0.603  0.841 |

The bold values indicate the statistically significant *P*-values (*P*<0.05)

^a^Significant after Bonferroni correction (*P*<2.4·10^-4^).

Abbreviations: *ABCB1*, ATP Binding Cassette Subfamily B Member 1
